# Supplementary material for: 2D/2D Heterojunction of TiO2 Nanoparticles and Ultrathin G-C3N4 Nanosheets for Efficient Photocatalytic Hydrogen Evolution
Source: Nanomaterials (Basel). 2022 May 4;12(9):1557. doi: 10.3390/nano12091557 (PMC9101109; doi:10.3390/nano12091557)
Supplement: Supplementary file 1 [file nanomaterials-12-01557-s001.zip › nanomaterials-1696458 supplementary.pdf]

Supporting Information

2D/2D Heterojunction of TiO_2 Nanosheets / Ultrathin g-C_3N_4 for efficient photocatalytic hydrogen evolution

Ruifeng Du[a,b], Baoying Li[c,*], Xu Han[d], Ke Xiao[a,b], Xiang Wang[a,b], Chaoqi Zhang[a,b], Jordi Arbiol[d,e], Andreu Cabot[a,e,*]

[a] Catalonia Energy Research Institute - IREC, Sant Adrià de Besòs, 08930 Barcelona, Spain

[b] Departament d'Enginyeria Electrònica i Biomèdica, Universitat de Barcelona, 08028, Barcelona, Spain

[c] Shandong Provincial Key Laboratory of Molecular Engineering, State Key Laboratory of Biobased Material and Green Papermaking, School of Chemistry and Chemical Engineering, Qilu University of Technology, Shandong Academy of Sciences, Jinan, 250353, P. R. China

[d] Catalan Institute of Nanoscience and Nanotechnology (ICN2), CSIC and BIST, Campus UAB, Bellaterra, 08193, Barcelona, Catalonia, Spain

[e] ICREA, Pg. Lluis Companys 23, 08010 Barcelona, Catalonia, Spain.

Characterization

The particle size and shape of the samples were characterized by scanning electron microscopy (SEM) using in a Zeiss Auriga microscope (Carl Zeiss, Jena, Germany) with an energy-dispersive X-ray spectroscopy (EDS) detector at 20 kV to study the composition and transmission electron microscopy (TEM) using a ZEISS LIBRA 120, operating at 120 kV. High-resolution TEM (HRTEM) studies were conducted using a field emission gun FEI Tecnai F20 microscope at 200 kV with a pointto-point resolution of 0.19 nm. Powder X-ray diffraction (XRD) patterns were collected directly from the as-synthesized nanoparticles dropped on Si(501) substrates on a Bruker AXS D8 Advance X-ray diffractometer with Nifiltered (2 µm thickness) Cu Kα radiation (λ = 1.5406 Å) operating at 40 kV and 40 mA. A LynxEye linear position-sensitive detector was used in reflection geometry. Characterization of the surface was done by X-ray photoelectron spectroscopy (XPS) on a SPECS system equipped with a XR50 source operating at 250 W and a Phoibos 150 MCD-9 detector. The pass energy of the hemispherical analyzer was set at 20 eV, and the energy step of high-resolution spectra was set at 0.05 eV. The pressure in the analysis chamber

was always below 10^{-7} Pa. The specific surface area and analysis of the pore size distribution were obtained from nitrogen adsorption/desorption isotherms on Tristar II 3020 Micromeritics system. Fourier transform infrared (FTIR) spectra were recorded on an Alpha Bruker spectrometer. The optical properties of samples were analyzed by a UV−vis spectrophotometer (UV-2600, Shimadzu). Photoluminescence (PL) spectra of aqueous photocatalysts suspension (0.1 g/L) were collected on a fluorescence spectrophotometer (F-7000, Hitachi), and the wavelength of excitation light is 370 nm. The decay time measurements were carried out on a compact fluorescence lifetime spectrometer (Quantaurus-Tau, C11367, HAMAMATSU), and an LED lamp (365 nm) was used as an excitation source.

Electrocatalysis measurement

The photocurrent and electrochemical impedance spectroscopy (EIS) investigations were carried out on a CHI-760 electrochemical analyzer using a three-electrode cell system with indium tin oxide (ITO)/sample as the working electrode, platinum net as the counter electrode and standard calomel electrode (SCE) as the reference. A 300 W Xe lamp with a cut-off filter of 420 nm was utilized as the light source. Furthermore, the ITO/sample electrodes were fabricated as follows: first, samples (5 mg) were added into solutions containing DI water (0.5mL), ethanol (0.5 mL) and Nafion (20uL) and ultrasonicated for 20 min. Then, the resultant sample slurry (0.1 mL) was casted onto pre-cleaned ITO glass and then dried at 60℃ for 2 h.

Apparent quantum efficiency (AQE) calculations

The apparent quantum efficiency can be evaluated from following equation:

$$\mathrm{AQE} = \frac{2 \times n_{H_2} \times N_A}{N}$$

$n_{\mathrm{H_2}}$ is the number of evolved H_2 molecules, N_A is Avogadro number (6.02×10^{23}) and N represents the number of incident photons, which can be calculated from following equation :

$$\mathrm{N} = \frac{\text{light intensity } (\mathrm{W\ cm^{-2}}) \times \text{illumination area } (cm^2)}{\frac{hc}{\lambda}}$$

h is plank constant (6.626×10^{-34} J·s = 4.136×10^{-15} eV·s), c is the speed of light (3.0×10^{8} m·s^{-1}), λ is the wavelength of light (380 nm, 420 nm).

Test method for light with wavelengths of 380 and 420 nm. The photocatalytic systems with 200 mg catalyst and 100 mL solution (90 mL DI water, 10 mL methanol and 1 wt% Pt cocatalyst). The mixed solution was bubbled with N_2 for 30 min to ensure anaerobic condition and illuminated 30

min with ultraviolet light before simulated solar light irradiation to measure H_2 evolution.The test time was 1h and irradiated area is 28.26 cm^2.

The light intensity and resulting n_{H_2} are listed in the table S2.

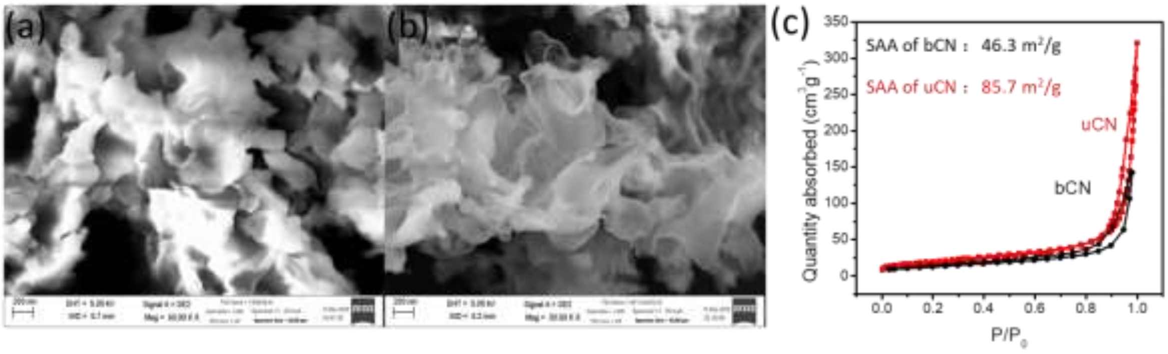

Figure S1. SEM image of (a) bulk g-C_3N_4 and (b) ultrathin g-C_3N_4, (c) N_2 adsorption-desorption isotherms of bCN and uCN.

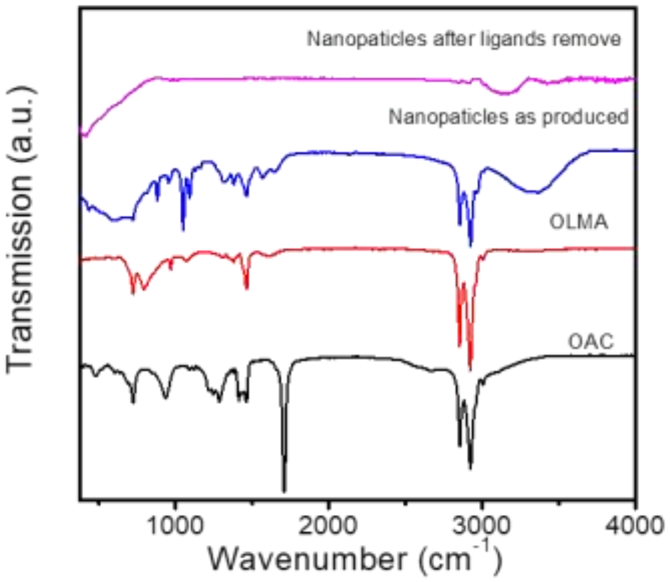

Figure S2. FTIR spectra of OAC, OLMA and TiO_2 before and after ligands remove.

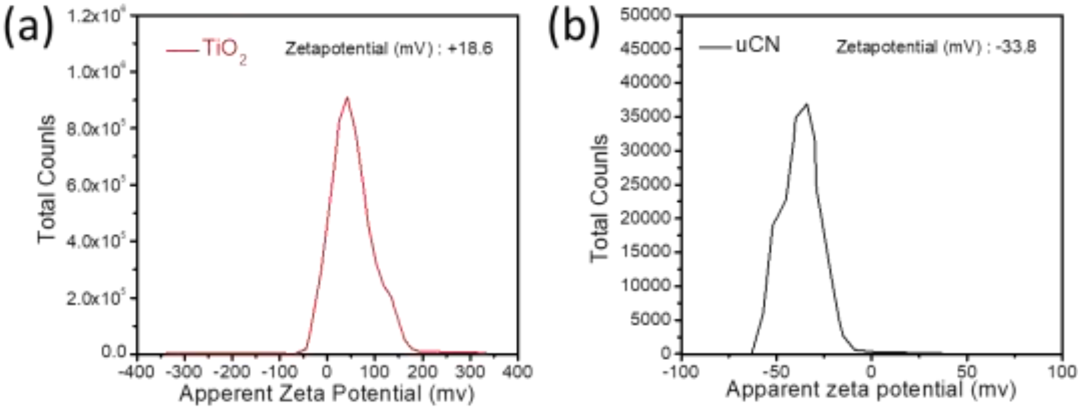

Figure S3. Zeta potential distribution spectrum of TiO_2 after ligands removal (a) and uCN (b).

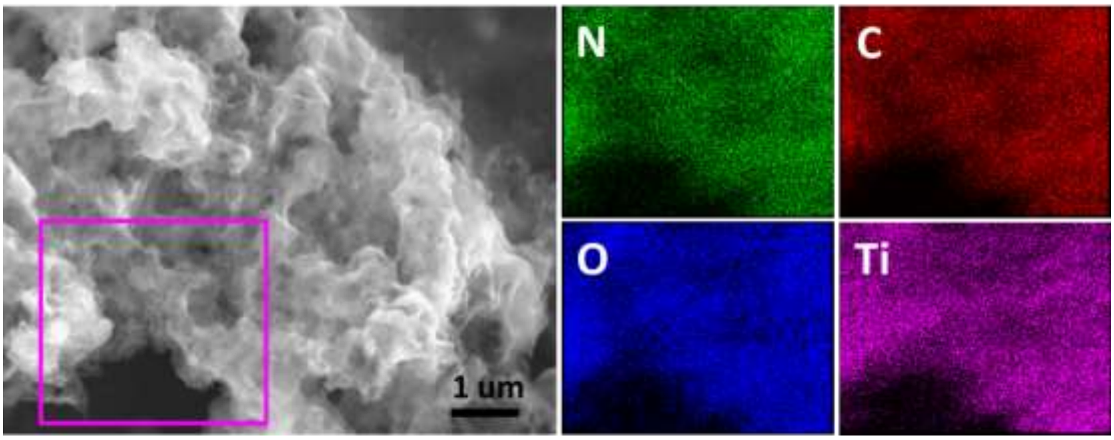

Figure S4. SEM image and EDS compositional maps of a T_1/uCN_1 composite

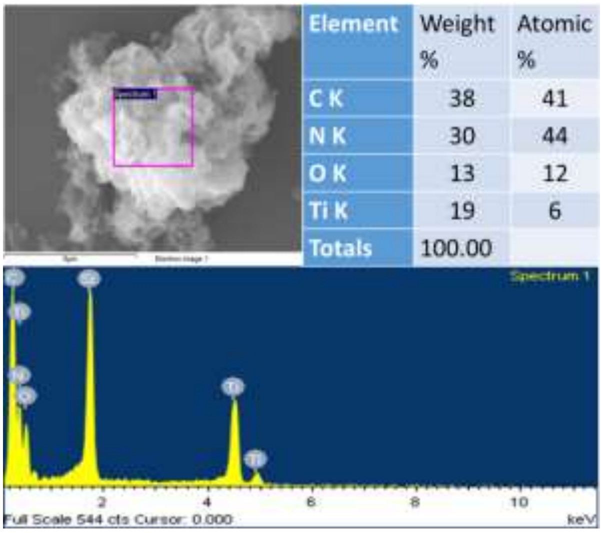

Element	Weight %	Atomic %
C K	38	41
N K	30	44
O K	13	12
Ti K	19	6
Totals	100.00	

Figure S5. SEM image of T1/uCN2 and corresponding EDS spectrum

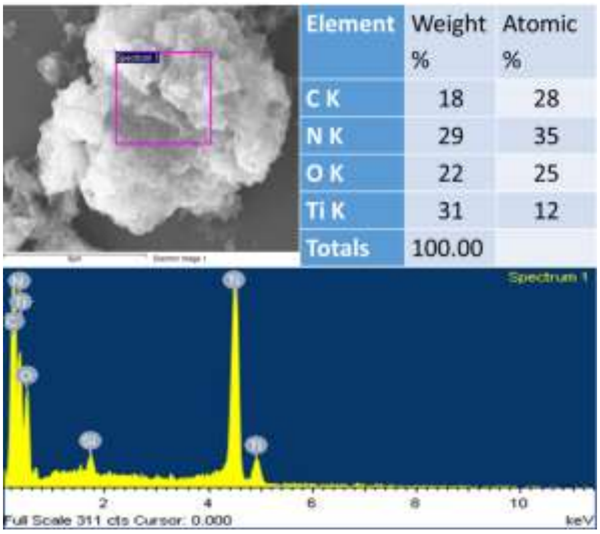

Element	Weight %	Atomic %
C K	18	28
N K	29	35
O K	22	25
Ti K	31	12
Totals	100.00	

Figure S6. SEM image of T1/uCN2 and corresponding EDS spectrum

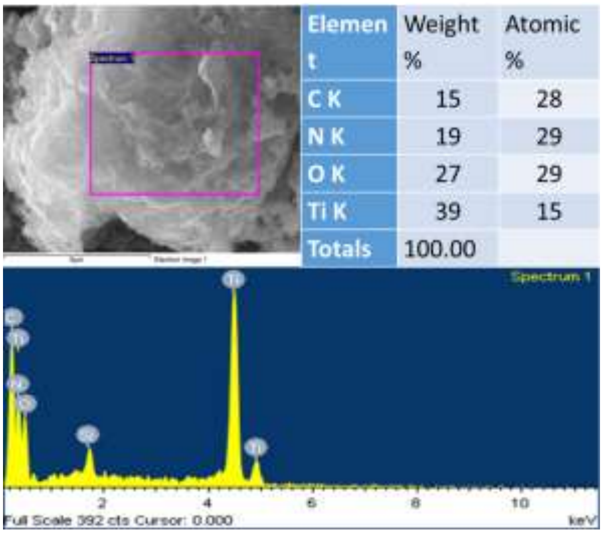

Elemen t	Weight %	Atomic %
C K	15	28
N K	19	29
O K	27	29
Ti K	39	15
Totals	100.00	

Figure S7. SEM image of T1/uCN2 and corresponding EDS spectrum

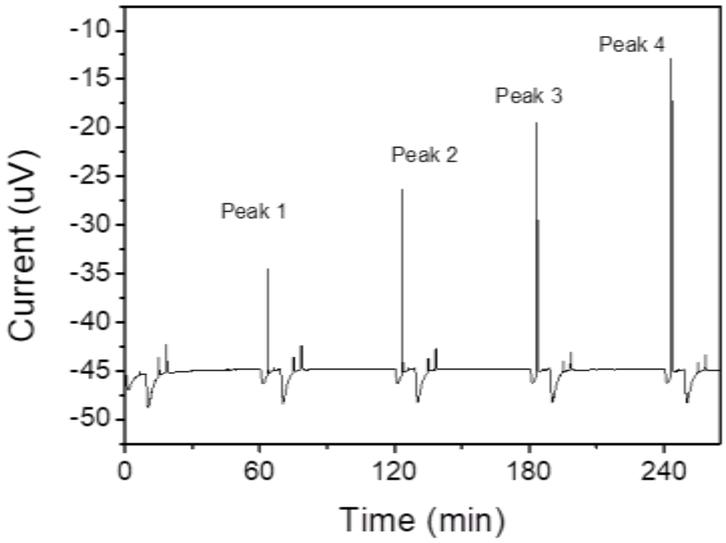

Figure S8. Chromatogram plots for 0.5 ml of standard hydrogen injected every half hour

Table S1. Gas Chromatography Peak Processing Data based on fig S8

Peak（#）	Peak time (min)	Peak height (uV)	Peak area (Uv*s)
1	61.760	10599	50777
2	121.765	18080	88649
3	181.771	28023	139827
4	241.786	36981	191326

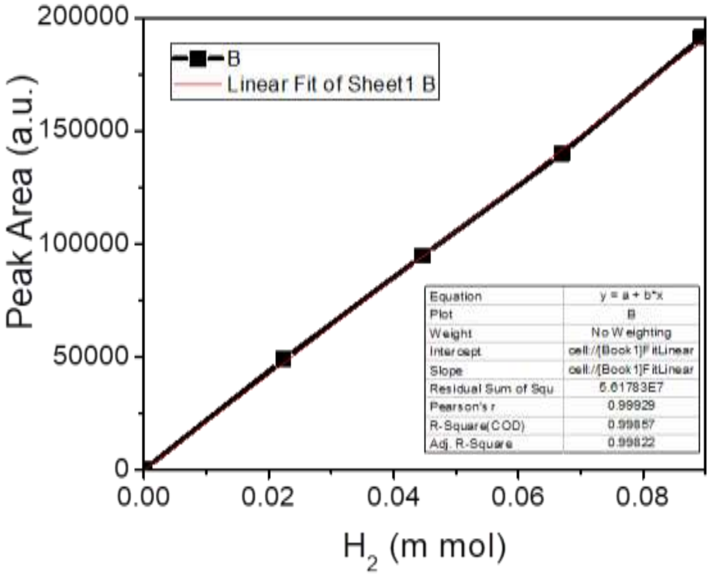

Figure S9. Standard hydrogen curve for gas chromatography

Table S2. Exponential decay-fitted parameters of fluorescence lifetime of uCN, TiO_2 and T_1/uCN_1

Sample	uCN	TiO_2	T_1/uCN_1
τ (ns)	3.51	3.15	4.72
τ_1 (ns)	1.04	0.96	1.49
τ_2 (ns)	6.03	5.47	6.93

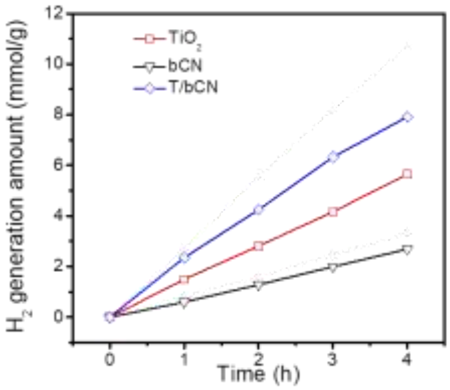

Figure S10. Photocatalytic hydrogen generation amount on bCN, TiO_2 and T_1/bCN_1 during 4 h under simulated solar light irradiation

Table S3. Photocatalytic hydrogen production about TiO_2/g-C_3N_4 based catalysts

**Photocatalyst**	**Additional co-catalyst**	**Light source**	**Activities (μmol h^{-1} g^{-1})**	**Sacrificial Agent**	**Ref.**
Ag/TiO_2/g-C_3N_4	1% Pt	400 nm filter	1707	TEOA	[1]
B-TiO_2/SiO_2/gC_3N_4	Pt	AM 1.5G filter	572.6	Methanol	[2]
MoS_2/g-C_3N_4/GO	No	AM 1.5G filter	1650	Na_2SO_3 (0.25 M).	[3]
TiO_2/g-C_3N_4	3% Pt	300 W	1820	TEOA	[4]
N-TiO_2/g-C_3N_4/NixP	No	300 W	5438	TEOA	[5]
NiS/TiO_2	Pt	300W	698	Lactic acid	[6]
Au/TiO_2-g-C_3N_4	No	150W	350	Methanol	[7]
g-C_3N_4/MMT/TiO_2	Pt	350 W	2213	Glycerol	[8]
ZnS- g-C_3N_4/TiO_2	No	300W	422	TEOA	[9]
Sheet TiO_2 / ultrathin g-C_3N_4	1% Pt	AM 1.5G filter	3875	Methanol	This work

Table S4. The AQE values with different incident light wavelengths for T_1/uCN_1

Wavelength (nm)	Light intensity (mW/cm^2)	T_1/uCN_1	
		n **H_2** (umol)	AQE (%)
380	4.51	58.3	7.16
420	12.14	55.8	2.67

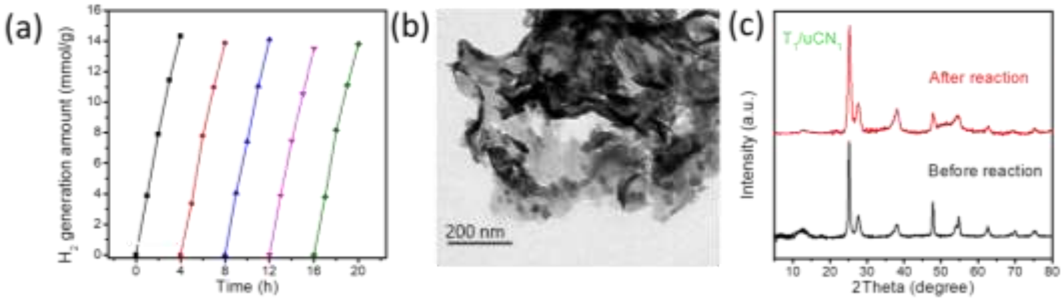

FFigure S11. (a) Stability cycles of the T_1/uCN_1 for H_2 evolution under simulated solar light irradiation; (b) TEM image of T_1/uCN_1 after 20 h photocatalytic H_2 evolution reaction and (c) XRD pattern of T_1/uCN_1 before and after 20 h photocatalytic H_2O_2 evolution reaction.

Reference

[1] R. Geng, J. Yin, J. Zhou, T. Jiao, Y. Feng, L. Zhang, Y. Chen, Z. Bai, Q. Peng, In Situ Construction of Ag/TiO2/g-C3N4 Heterojunction Nanocomposite Based on Hierarchical Co-Assembly with Sustainable Hydrogen Evolution, Nanomater. . 10 (2020). doi:10.3390/nano10010001.

[2] L. Shen, Z. Xing, J. Zou, Z. Li, X. Wu, Y. Zhang, Q. Zhu, S. Yang, W. Zhou, Black TiO2 nanobelts/g-C3N4 nanosheets laminated heterojunctions with efficient visible-light-driven photocatalytic performance, Sci. Rep. 7 (2017) 1–11.

[3] M. Wang, P. Ju, J. Li, Y. Zhao, X. Han, Z. Hao, Facile synthesis of MoS2/g-C3N4/GO ternary heterojunction with enhanced photocatalytic activity for water splitting, ACS Sustain. Chem. Eng. 5 (2017) 7878–7886.

[4] W. Gu, F. Lu, C. Wang, S. Kuga, L. Wu, Y. Huang, M. Wu, Face-to-face interfacial assembly of ultrathin g-C3N4 and anatase TiO2 nanosheets for enhanced solar photocatalytic activity, ACS Appl. Mater. Interfaces. 9 (2017) 28674–28684.

[5] M. Wu, J. Zhang, C. Liu, Y. Gong, R. Wang, B. He, H. Wang, Rational Design and Fabrication of Noble-metal-free NixP Cocatalyst Embedded 3D N-TiO2/g-C3N4 Heterojunctions with Enhanced Photocatalytic Hydrogen Evolution, ChemCatChem. 10 (2018) 3069–3077.

[6] L. Zhang, B. Tian, F. Chen, J. Zhang, Nickel sulfide as co-catalyst on nanostructured TiO2 for photocatalytic hydrogen evolution, Int. J. Hydrogen Energy. 37 (2012) 17060–17067.

[7] C. Marchal, T. Cottineau, M.G. Méndez-Medrano, C. Colbeau-Justin, V. Caps, V. Keller, Au/TiO2–gC3N4 nanocomposites for enhanced photocatalytic H2 production from water

under visible light irradiation with very low quantities of sacrificial agents, Adv. Energy Mater. 8 (2018) 1702142.

[8] N. Fajrina, M. Tahir, 2D-montmorillonite-dispersed g-C3N4/TiO2 2D/0Dnanocomposite for enhanced photo-induced H2 evolution from glycerol-water mixture, Appl. Surf. Sci. 471 (2019) 1053–1064.

[9] C. Zhang, Y. Zhou, J. Bao, J. Fang, S. Zhao, Y. Zhang, X. Sheng, W. Chen, Structure regulation of ZnS@ g-C3N4/TiO2 nanospheres for efficient photocatalytic H2 production under visible-light irradiation, Chem. Eng. J. 346 (2018) 226–237.
